# The “3Ds” of Growing Kidney Organoids: Advances in Nephron Development, Disease Modeling, and Drug Screening

**DOI:** 10.3390/cells12040549

**Published:** 2023-02-08

**Authors:** Brooke E. Chambers, Nicole E. Weaver, Rebecca A. Wingert

**Affiliations:** Department of Biological Sciences, Center for Stem Cells and Regenerative Medicine, Center for Zebrafish Research, Boler-Parseghian Center for Rare and Neglected Diseases, Warren Center for Drug Discovery, University of Notre Dame, Notre Dame, IN 46556, USA

**Keywords:** kidney, nephron, organoid, stem cell, nephron progenitor, zebrafish

## Abstract

A kidney organoid is a three-dimensional (3D) cellular aggregate grown from stem cells in vitro that undergoes self-organization, recapitulating aspects of normal renal development to produce nephron structures that resemble the native kidney organ. These miniature kidney-like structures can also be derived from primary patient cells and thus provide simplified context to observe how mutations in kidney-disease-associated genes affect organogenesis and physiological function. In the past several years, advances in kidney organoid technologies have achieved the formation of renal organoids with enhanced numbers of specialized cell types, less heterogeneity, and more architectural complexity. Microfluidic bioreactor culture devices, single-cell transcriptomics, and bioinformatic analyses have accelerated the development of more sophisticated renal organoids and tailored them to become increasingly amenable to high-throughput experimentation. However, many significant challenges remain in realizing the use of kidney organoids for renal replacement therapies. This review presents an overview of the renal organoid field and selected highlights of recent cutting-edge kidney organoid research with a focus on embryonic development, modeling renal disease, and personalized drug screening.

## 1. Introduction

Organoids are products of recent advances in stem cell biology and 3D tissue culture research, where miniaturized structures that resemble human organs can be grown in vitro from stem cells [1]. Generating these mini-organs begins with the culturing of human embryonic stem cells (ESCs), induced pluripotent stem cells (iPSCs), or tissue-resident adult stem cells (ASCs), which, when exposed to the correct nutrients, growth factors, and instructive signals, are induced to undergo self-organization and differentiation [2]. The ability to recapitulate aspects of human development in vitro marks a major milestone, as nearly all of our current understanding of embryogenesis has been drawn from landmark studies conducted in animal models [3]. Not only does the study of human organoids help address gaps in developmental biology knowledge related to species divergence, but it offers substantial opportunities for disease modeling and preclinical studies [4]. To this point, organoids derived from an individual’s cells may offer a scalable system for personalized drug screening and show the potential to enhance cellular therapies and tissue grafts [5].

The surge of interest in organoid research began about fifteen years ago when labs began reporting the ability to grow ‘in a dish’ miniature versions of the mammalian eye [6,7,8], the intestine [9,10], and the brain [11]. The sensation has only expanded over time as researchers successfully created methods to grow organoids of the liver, stomach, retina, prostate, lung, and kidney, among others [12,13]. Here, we provide a broad overview of kidney organoid technology, discuss major challenges facing the application of renal organoids in basic and clinical research endeavors, and explore exciting advances in three areas: development, disease, and drug discovery.

## 2. Kidney Organoids Parallel Early Metanephric Development and Can Be Generated by Several Distinct Protocols

The adult kidney is believed to exhibit the highest complexity regarding cellular composition aside from the central nervous system, as it encompasses greater than 25 distinct cell types that are arranged in an intricate tissue architecture [14]. Together, this diverse medley of cells accomplishes a rich array of physiological tasks, including waste and toxin excretion; regulation of fluid and ion homeostasis, which maintains blood osmolarity; regulation of blood volume and, thereby, blood pressure; regulation of blood pH; and hormone production of erythropoietin and calcitriol to regulate red blood cell production and calcium homeostasis, respectively [15,16,17,18,19,20,21,22,23,24]. Signaling cues and molecular crosstalk during embryonic development are responsible for fueling the complexity of induction, patterning, and differentiation events that enable the production of a diversity of renal cell programs [25,26,27,28].

As with other organs, our understanding of renal development (as well as disease) has been largely informed by research in animal models, ranging from invertebrates such as the fruit fly and nematode to vertebrates such as the frog, zebrafish, chick, quail, and mouse [29,30,31,32]. There is an increasing appreciation of conserved and divergent features from ongoing work to assess human kidney development [33,34,35,36,37,38,39]. From many decades of study, activities of the Wnt (Wingless/Int-1), bone morphogenetic protein (BMP), fibroblast growth factor (FGF), and retinoic acid (RA) signaling pathways have been identified as being responsible for driving the underlying molecular events that lead to the elaboration of the kidney [40].

With this perspective in mind, the current working model is that kidney development in mammals begins with the formation of the primitive streak, which gives rise to the intermediate mesoderm (IM) through sequential exposure to Wnt and BMP activity [39,40]. The IM is regionalized to form two progenitor pools: the nephric duct from the anterior IM and the metanephric mesenchyme (MM) from the posterior IM [39,40]. These two renal progenitor populations undergo reciprocal inductive signaling, which elaborates an intricate organ architecture [39,40]. The reciprocal cross-talk causes the nephric duct to form a ureteric bud (UB), which grows and undergoes reiterative branching morphogenesis to create an arborized collecting duct system [39,40]. Concurrently, the metanephric mesenchyme associated with the branch points condenses to form cap mesenchyme, and these nephron progenitor cells (NPCs) undergo epithelialization, patterning, and differentiation to form the specialized cellular compartments of the nephron tubule [39,40]. Indeed, Wnt signaling from the UB is the trigger for the mesenchymal to epithelial (MET) transition of NPCs [40]. Glial-derived neurotrophic factor (GDNF) from the MM signals UB proliferation, subsequent growth, and then dichotomous branching to make the ureteric epithelial tree [40].

Several molecular triggers to make NPCs into functional nephron units come from the ureteric branch tips [40]. From this source, NPCs are maintained in part by FGF9, FGF20, BMP7, and low canonical Wnt signaling. Conversely, NPCs undergo a MET when exposed to higher levels of canonical Wnt signaling via Wnt9b and Wnt4. This MET transforms a ‘pre-tubular’ aggregate of mesenchyme into a renal vesicle, which is an epithelial structure that elongates into a ‘comma shaped’ and then an ‘S-shaped body’ and ultimately differentiates into a nephron. Nephrons are the functional unit of the kidney that have a series of unique functional domains, known as segments, along the length of their proximal-distal axis, including a glomerulus (blood filter) at the proximal-most end followed by a series of discrete ion-transporting tubule segments (proximal, intermediate/loop of Henle, distal), which merge distally with the collecting duct system for waste excretion [14]. This discrete nephron segmental organization is established in part by a gradient of Wnt signaling, a RA morphogen gradient, and regionalized Notch signaling—a combination that is interestingly well conserved across species [40].

Further complexity of the kidney lies in the cellular components that reside in the stroma that surrounds the arborized arrangement of nephrons that are organized around the central collecting system. Each nephron is surrounded by an extensive capillary system as well as stromal (interstitial) cells with various functions, like immune cells [14]. In humans, nephrogenesis continues until approximately 36 weeks of gestation and then abruptly ceases. Thus, the so-called ‘endowment’ of nephrons at birth is the lifelong source of renal function, and humans born prematurely with lower nephron endowments are at higher risk for kidney disease [41,42].

Protocols to generate kidney organoids mimic the order of major events involved in mammalian metanephric development by exposing ESCs, iPSCs, embryonic kidney progenitors, or adult cells to a sequence of defined factors to create a normal developmental sequence, but in vitro [43,44,45,46,47,48]. Success has been achieved using defined factors either singly or in combination, with varied orders of exposure, duration, and of course, concentration [43,44,45,46,47,48]. The sequential exposure to the “right” recipe of signals accomplishes IM specification and regionalization, followed by the formation of nephron progenitor cells (NPCs), NPC derivatives such as nephrons, stromal cells, UB-like cells, and endothelial cells over the course of 2–4 weeks [43,44,45,46,47,48]. Initially, there were several main methods of generating kidney organoids from stem cell sources based on innovative and enterprising work of researchers from the Little lab [49,50,51], Nishinakamura lab [52,53], Izpisua-Belmonte [54,55,56], and Bonventre lab [57,58,59].

There has been continued ground-breaking work by these labs and others to identify enhanced methods for renal organoid cultivation [60,61,62,63]. For example, there have been advances to make protocols simpler and more scalable [64,65,66], to achieve automation [67], and to generate increasingly more complete kidney structures [68,69]. Indeed, dozens of reports now describe methodical improvements in controlling nephrogenesis, particularly with regard to lineage specification and segment patterning [69,70,71,72,73,74,75,76,77,78,79,80,81,82,83,84,85,86,87,88,89,90,91,92,93,94,95,96,97,98,99,100,101,102,103,104,105,106]. We refer readers to several useful contemporary reviews for a very comprehensive methodological comparison of these existing protocols [61,62,69]. In the following section, we provide an overview of the major current limitations that still plague renal organoid methodologies, then proceed to explore three exciting research foci which are unleashing new powerful opportunities to further improve renal organoids and promote their medical application.

## 3. Ongoing Limitations in Renal Organoid Research

**Figure 1:**
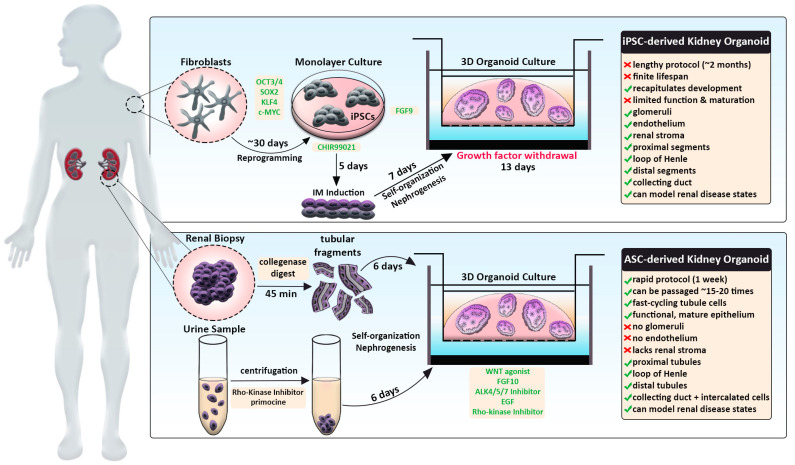
Schematic comparing human iPSC-derived and ASC-derived kidney organoid strategies. iPSC-derived kidney organoid protocol (**top**) typically entails reprogramming patient fibroblasts (grey), induction of IM in monolayer culture conditions and directed differentiation by timed addition of certain factors (green). This process is relatively lengthy but more accurately recapitulates processes taking place during embryonic kidney development. ASC-derived kidney organoid protocol (**bottom**), also termed ‘tubuloids,’ involves the isolation of renal cells via biopsy or urine sample (purple). Upon processing, tubular fragments or urine-derived cells are directly seeded into 3D culture conditions and treated with growth factors (green) to induce rapid differentiation of nephron tubule structures. Resulting tubuloids constitute mature epithelium, can be passaged many times, but lack certain kidney cell types. Both systems promote the 3D assembly of organoids by employing Matrigel-coated transwell culture apparatuses.

Traditional kidney organoid protocols begin with growing human iPSCs in a monolayer, or two-dimensional (2D) culture, for 7 days to induce intermediate mesoderm by the addition of two main molecules: CHIR99201 and FGF9 [61,62,107]. After a week of monolayer culture, the cells are dissociated, plated in 3D culture conditions, and subjected to APEL media supplemented with FGF9 and heparin. Within the next few days, the cells will aggregate, self-organize, and undergo nephrogenesis (Figure 1, top panel). From day 12 on, the maturing kidney organoids are subjected to media without FGF9 and heparin. This protocol produces ‘mini-kidneys’ that have up to several hundred nephron-like structures with multiple cell lineages, including glomeruli, tubule segments (e.g., proximal, loop of Henle, distal), and various stromal cells.

In addition, kidney organoids that are termed ‘tubuloids’ can be produced easily and rapidly by culturing ASCs obtained from a renal biopsy (Figure 1, bottom panel). By comparison, tubuloids lack glomeruli, endothelium, and stroma but contain proximal, intermediate, and distal segments along with collecting ducts. In Section 6, we discuss tubuloids in further detail and how they offer many new future opportunities, such as the creation of resources like biobanks to facilitate personalized medicine. First, in the sections below, we will discuss the current limitations shared by both protocols that pose challenges for using organoids to study development and disease-associated alterations.

### 3.1. Technical Challenges of Reproducibility and Cellular Heterogeneity

Both kidney organoid protocols generate renal organoids that exhibit variations in cell type composition, as briefly described in the previous section. Interestingly, within a given experiment using any of the established protocols to grow renal organoids, the renal organoids are highly similar. However, there is high technical variability or so-called ‘batch-to-batch’ variation between different experiments using the same protocol. This is thought to partly reflect differences in the groups of reagents used, such as growth factors. There are also variability differences based on the cell line source and even from different stocks of the same cell line. Current protocols also can elicit different total percentages of nephron segment populations, e.g., more tubular cells. Thus, it remains a challenge how to tailor conditions in the right manner to create nephrons with the proper segment ratios.

### 3.2. Architectural Simplicity and Abnormal Nephrogenesis

Renal organoids display notable anatomical and compositional differences compared to the naturally developed mammalian kidney. At birth, the final human kidney form encompasses from ~200,000 to upwards of 1–2 million nephrons arranged in cortical and medullary zones, with each individual nephron being connected on one end to the systemic vasculature and the other to a central, arborized collecting duct system [41,42]. The elaborate structure of the human kidney develops over the course of approximately 200 days [26]. In contrast, most kidney organoid protocols occur over a 2–4-week time span.

For the most part, renal organoids have lacked a higher order/complex organization, with nephrons randomly arranged and intermingled, sometimes with various overt malformations such as nephron-nephron connections and branched nephrons. However, some protocols have been able to foster the cultivation of a clearly defined ureteric bud/collecting duct-like structure [105].

### 3.3. Generation of Off-Target Cell Types, While Some Renal Cell Types Are Missing Altogether

In addition to the heterogeneity of renal organoids, current methods produce nephrons and surrounding interstitium with missing or underrepresented cell types. For example, macrophages and other immune cells are absent, as well as mesangial cells within glomeruli [61,62,107]. Renal organoids lack vasculature, which limits how much they can grow in vitro. Further, they contain varying percentages of ‘off-target’ populations such as neurons, skeletal muscle, satellite cells, and melanocytes, which expand over prolonged culture times [61,62,107].

### 3.4. Renal Organoids Show Limited Lifespan and Lack Stem/Progenitor Self-Renewal Characteristics

In addition to reduced nephron numbers, kidney organoids have a finite lifespan with limited nephrogenesis capability. To date, the interval over which nephrogenesis occurs in these cultures is also quite limited. During the culture process, there is a single bout of nephron formation but no ongoing rounds over time. The growth conditions have not supported the emergence of the proper microenvironment for the lengthy survival and/or self-renewal of NPCs. In part, this barrier likely exists because of differences in the stromal cell populations within current renal organoids. In support of this notion, a recently reported induced stroma protocol with mouse ES cells has supported the generation of more advanced higher-order organoids [105]. Nevertheless, escalating nephron numbers within culture remains an aspect to address.

The limited culture longevity of realized renal organoids is also impacted by the absence of functional vasculature in this setting. So far, the best methods to achieve vascularization have been to transplant the renal organoid into another host system, such as a mouse, to co-culture on the chick chorioallantoic membrane or to culture under sheer stress in a microfluidic device [53,71,75,76,77,78,80,87,93,108]. Interestingly, transplanted human renal organoids are vascularized through angiogenesis of murine host cells [76,95]. Such organoids can undergo successful blood perfusion and glomerular filtration [76,95].

### 3.5. The Renal Organoid ‘Fetal State’—A Lack of Maturity in Renal Cell Types

Nephrons in renal organoids also exhibit features of an immature fetal state which has been likened to the first or second-trimester human fetal kidney [50,109,110] and shows limited functional characteristics. For example, proximal tubules produced via organoid culture express decreased levels of anionic, amino acid, and glucose transport proteins, which are inherent for the physiological function of this nephron compartment. However, a recent report has shown enhanced mature features by inhibition of Wnt signaling [110]. Further, there has been remarkable progress in achieving the recipe for cultivating the pattern formation and subsequent differentiation of some structures and their composite lineages—such as the 3D glomeruli with podocytes and parietal epithelial cells that form the Bowman’s capsule [111]. Podocytes display hallmark differentiated features: ultrastructural traits such as foot processes, gene expression characteristics such as collagen and laminin switching, and autonomous calcium signaling [50,72,87,89,112]. Though, as previously noted, mesangial cells are absent within in vitro conditions.

While kidney organoids are a simplified representation of the human kidney, many researchers continue to improve these models by employing innovative technologies such as single-cell RNA sequencing and microbioreactors to optimize differentiation protocols. In the following sections, we discuss how the application of several such technologies has led to advancements in the kidney organoid model.

## 4. Single-Cell Transcriptomics: Helping to Generate Reproducible Kidney Organoids

Single-cell sequencing is being used to comprehensively identify the variety of cell types which emerge in kidney organoid cultures [108,109,112] and to better understand human kidney development [33,34,35,36,37,38]. The intersect of these emerging datasets has been leveraged progressively to gain tremendous insights into the molecular characteristics of renal organoids, leading to new understandings about their variability within and between research labs. For example, work conducted by Phipson et al., 2019 collected a comprehensive transcriptional and morphological profile of organoids from the same human iPSC source [113]. Their experimental design involved six separate differentiation experiments (batches) that were each performed up to 12 months apart with different reagent lots, culture media, and recombinant growth factors. Because most nephron structures typically form by day 18, the researchers chose this time point to examine ‘batch-to-batch’ organoid variation. Transcriptional profiling revealed day 18 organoids within the same batch clustered closely with one another, but significant variation across different experimental batches occurred. Genes that exhibited the highest degree of differential expression were mostly identified as nephron maturation markers. For example, the expression of podocyte differentiation genes NPHS1, NPHS2, and PTPRO were highly variable. The researchers also employed single-cell RNA-sequencing technology to examine shifts in differentiated cell populations across experimental batches. Most cell clusters were present in all organoids generated; however, relative proportions of these cell types significantly varied between batches. Surprisingly, when the authors examined variations between different iPSC lines within the same batch, the transcriptomic profiles and population clusters were remarkably analogous. Altogether, this data prompted the authors to develop a new standardized method to predict relative kidney organoid ‘age.’ Transcriptional analysis of differentiating organoids from day 7 to 25 was used to identify markers of nephron maturity that exhibited the tightest linear correlation during this timeframe. Importantly, Phipson et al., 2019 identified 10 biomarkers that can be utilized to accurately predict relative organoid maturity [113].

This method could have broad-reaching impacts in the field regarding the normalization of variation across kidney organoid models. As proof of principle, the authors applied their strategy for predicting relative organoid age and highly variable gene lists to improve transcriptional analysis of healthy versus diseased kidney organoids [113]. For this experiment, diseased kidney organoids were generated from a patient with Mainzer-Saldino syndrome possessing mutations in IFT140, which disrupts primary cilia and causes nephronophthisis. The authors’ predictive biomarker strategy revealed that patient-derived renal organoids are ‘younger’ and exhibit an immature signature compared to healthy gene-corrected controls. Additionally, upon differential gene expression analysis, the removal of highly variable confounding genes brought to light more biologically relevant pathways. Subtracting this noise enriched pertinent gene ontology terms such as ‘plasma membrane region’ and ‘apical part of the cell,’ which more accurately depicts the patient’s ciliopathic disease state. Taken together, this study illustrates that batch-to-batch differences are the greatest drivers of temporal variability; therefore, moving forward, it is crucial that future organoid studies take this into account when comparing data across experimental groups. This study combats significant challenges the field is facing regarding accurate disease modeling and presents useful practices to standardize the comparison of patient and control renal organoids. Interestingly, single-cell transcriptomics has also revealed that human renal organoids transplanted under the kidney capsule of immunocompromised mice exhibited diminished off-target cells [114], suggesting improved organoid quality along with likely maturation of the organoid to a more mature state.

Continued profiling of the fetal mouse and human kidney, e.g., [115,116], has continued to support efforts to improve renal organoids. Most recently, an analytical tool was created to enable better cross-comparisons of renal organoids, which will be especially useful as new adjustments are made with existing protocols or even as entirely novel approaches are realized [117].

## 5. Microfluidic Bioreactors: A Perfusion Platform to Culture Kidney Organoids

Tools known as microfluidic bioreactors are an emerging technology that is showing promise in enhancing both renal organoid differentiation and cell type. Traditionally, microfluidic platforms have been used in a broad range of scientific applications such as single-cell studies, filtration, small molecule screens, organ-on-chip, etc. As of recent, this strategy has been applied to tumor cell biology and stem cell biology to enhance 3D cell culture methods. In this context, microfluidic bioreactors serve as miniaturized culturing vessels that create a microenvironment that better recapitulates in vivo conditions. Oftentimes, scaffolds are embedded within the microfluidic platform to allow the continual diffusion of key nutrients and secreted factors, which helps mimic dynamic physiological conditions. Other advantages of this system include automated control of nutrient exchange, waste removal, chemical gradients, temperature, oxygen levels, and flow rate [117]. Because these bioreactors are highly amenable to automation, this tool could help standardize the generation of kidney organoids and minimize variation amongst research studies. As mentioned previously, physical cues were found to enhance the growth and maturation of kidney organoids: they exhibited enhanced glomerular vascularization and morphogenesis; tubules possessed enhanced cellular polarity and expressed more mature gene expression profiles [80].

Because conventional kidney organoid protocols involve static cell culture practices, it hampers the ability to assess spatiotemporal, combinatorial, and paracrine effects on cell fate. To address these multifaceted issues, Glass et al., 2020 employed microfluidic bioreactors to allow continuous perfusion of media and secreted factors during renal patterning [90]. In essence, renal organoids were generated by treating a human embryonic stem cell line (HES3) with CHIR and Fgf9 in a monolayer culture for 6 days. Cells were then seeded in microbioreactors for an additional 3 to 6 days for further experimentation. The researchers developed a microbioreactor-based assay (MBA) that incorporates 10 serially connected wells across 27 rows allowing for the analysis of 270 unique conditions and dissemination of secreted paracrine factors from upstream wells. Using this setup, a factorial screen of materials, including WNT (CHIR), FGF9, RA, and BMP7, was conducted to survey the effect of these morphogens on early renal cell specification. After the organoids were subjected to MBA perfusion conditions, image cytometry for three markers (GATA3, WT1, and ECAD) provided a high-throughput readout for ureteric epithelium, metanephric mesenchyme, early proximal nephron, early distal nephron, and stromal progenitor populations.

Paracrine-mediated changes in cell fate were observed in the first column of every MBA, where no input factors were added to the media; however, alterations in cell patterning were evident in downstream wells [90]. Their factorial screen also revealed a short pulse of WNT activation combined with FGF9 swayed cells toward ureteric epithelium and early distal nephron fates and inhibited the metanephric mesenchyme at high concentrations. However, by day 12, intermediate FGF concentrations supported metanephric mesenchyme development. These findings highlight the spatiotemporal sensitivity of developing organoid structures to FGF dosage. Additionally, BMP7, in the presence of FGF9, biases organoids to form early proximal nephron structures. Contrary to previous accounts, RA treatments did not result in significant alterations in MBA-based organoid differentiation. Lastly, prolonged WNT activation elevated ureteric and distal nephron cell numbers.

Glass and colleagues (2020) MBA system demonstrates how upstream wells condition the microenvironment and initiate downstream shifts in renal lineages [90]. These elegant experiments divulge a clear role for paracrine signaling that has remained unappreciated in static organoid culture systems. Because the fraction of proximal or distal nephron fates can be tuned via specific FGF9, BMP7, and CHIR regimens, this information could prove valuable for future organoid studies to more accurately recapitulate human kidney organogenesis. Along those lines, their high-throughput MBA screen produced wells that achieved near-pure renal cell specification. These near-perfect conditions could optimize multicellular differentiation protocols and diminish the creation of off-target cell types, as these problems have been commonly reported in previous kidney organoid initiatives. Interestingly, MBA-generated organoids were only cultured for a total of 12 days, as compared to traditional protocols that are carried out for approximately 30 days. Although this study addresses early nephron patterning, it does not track the subsequent maturation of these cells. Extending the timeline of this MBA approach may hold promise in improving renal differentiation status to generate mature, functional epithelium that faithfully reproduces in vivo kidneys. In sum, this study provides an excellent example of how the power of MBA technology can be harnessed to modulate exogenous and paracrine signaling molecules to home in optimal conditions to produce kidney organoids with specific proportions of cell populations.

## 6. Disease Modeling from Kidney Organoids to ‘Tubuloids’

Greater than 300 genes are implicated in the origins of kidney disease [118] and span a broad spectrum of disorders, including acute kidney disease (AKI), Alport Syndrome, nephrotic syndrome, tubulopathies, polycystic kidney diseases (PKD), chronic kidney disease (CKD), and congenital anomalies of the kidney and urinary tract (CAKUT), among others [119,120,121,122,123,124,125,126,127,128,129]. To date, there are limited therapies available for these conditions, coupled with a poor understanding of genotype-phenotype correlations [119,120,121,122,123,124,125,126,127,128,129]. While our present knowledge about renal diseases has benefited immensely from years of research using animal models, many gaps remain, and not all aspects apply to humans due to species variation and their substantially higher complexity [30,31,32,33,34,35,36,37,38,39,130,131,132,133,134].

Fortunately, the advent of cellular reprogramming and organoid techniques has catalyzed the modeling of human cells and, thereby, patient-specific conditions ‘in a dish’ [135]. As described in the previous sections, organoid technologies are continually evolving to more faithfully replicate kidney development. Because a significant portion of genetic kidney diseases are initiated by disruptions in developmental processes, such as patterning, differentiation, and maturation, renal organoids are positioned as a unique tool to investigate disease etiology and pathogenesis. Thus, kidney organoids are gaining traction in the field as a translatable model for renal diseases due to inborn human genetic lesions as well as acquired injuries, such as exposure to nephrotoxins.

Research conducted by Low and colleagues (2019) demonstrates how autosomal recessive polycystic kidney disease (ARPKD) can be modeled in renal organoids [84]. The investigators reprogrammed fibroblasts from an ARPKD patient with a c.11630delT mutation for further organoid experimentation. In parallel, CRISPR-corrected iPSC lines were generated from the same source to serve as an isogenic control group. Intriguingly, when subjected to 3D culture, both control and ARPKD iPSCs self-organized and formed patterned organoid structures at similar efficiencies. Following extended culture, only a small percentage of ARPKD organoids underwent spontaneous cyst development. This phenomenon is potentially explained by the ‘two-hit theory’ of PKD progression, where a genetic mutation in combination with a secondary environmental or mutagenic insult is required to initiate cystogenesis [136].

To address this issue, the researchers supplied a secondary insult by challenging kidney organoids with forskolin or 8-Br-cAMP treatments to upregulate intracellular cAMP activity [84]. Upon treatment, ARPK organoids exhibited significant cystogenesis as compared to controls. These cystic organoid phenotypes consisted of expanded tubule lumens and reduced expression of segment-specific nephron markers. Additionally, the proximal tubules were rendered nonfunctional, as indicated by their inability to uptake fluorescent-conjugated dextran. The researchers then evaluated the effects of treating their new ARPKD organoid model with two different chemical compounds, Thapsigargin or CFTR inhibitor, which were previously established to mitigate cystogenesis. As expected, both treatments successfully blocked cyst formation. Overall, their experiments provide proof that principle organoids can effectively model clinical features of ARPKD. This investigation merely scratches the surface regarding the potential of kidney organoids as a platform for personalized drug screening and provides a solid foundation for exciting follow-up studies. In this light, researchers have generated a “renal biobank” of organoids derived from children with various childhood cancers [136].

Additionally, research from Freedman and colleagues has provided new insights into the role of the microenvironment during PKD using kidney organoids derived from *PKD1*^−/−^ or *PKD2*^−/−^ human pluripotent stem cells [58]. Researchers found several biomaterials that impact cyst growth, such as adherence cues, whose removal was shown to promote cystogenesis [137]. In contrast, conditions that strengthened the stromal compartment favored migratory repair in lieu of cyst formation [137]. These studies provide compelling evidence that biomaterials have crucial impacts on the formation of tubule cysts.

Up until this point, the studies described in this review have derived kidney organoids from either an iPSC source or a human ESC line (Figure 1, top panel). There are caveats in applying the iPSC method to model clinical diseases in a personalized approach. First, the workflow is relatively lengthy, as the reprogramming of primary patient cells and directed differentiation steps can take up to several months. Moreover, iPSC-derived kidney organoids have a finite lifespan and a limited capacity for expansion in culture. Lastly, this model recapitulates developmental processes and struggles to achieve terminal differentiation but can provide a valid system to study congenital diseases.

As an alternate method, an ASC-derived kidney organoid model termed ‘tubuloids’ has been formulated that can be generated within one week, expanded for about 20 passages, and remain genetically stable (Figure 1, bottom panel) [86,97]. The protocol consists of digesting cortical kidney resections with collagenase, seeding tubule fragments in Matrigel, and culturing in conditioned media containing WNT agonist, FGF10, ALK4/5/7 inhibitor, EGF, and Rho-kinase inhibitor (Figure 1). Within 6 days, cystic conglomerates formed that encompassed fast-cycling, epithelialized nephron tubule structures. Transcriptomics indicated that several nephron compartments were present in tubuloids: proximal tubule (ABCC1/3/4, SLC22A3, SLC40A1), loop of Henle (CLDN10, CLDN14), distal tubule (PCBD1, SLC41A3), and collecting duct (CDH1, GATA3, AQP3). Importantly, single-cell RNA-sequencing of tubuloids detected a unique cluster expressing intercalated cell signature genes, which is a highly underrepresented collecting duct cell populace in other kidney organoid systems [113]. Additionally, tubuloids contained putative tubule progenitor clusters that lacked expression of segment-specific genes. In accordance with this observation, tubuloids possess cells with multilineage potential, as the researchers were able to generate a clonal line that gave rise to multiple nephron segments from a single cell.

Further, tubuloids can faithfully model an assortment of kidney disease states [86]. The system effectively recapitulated BK viral infection and presented clinical features like enlarged tubular nuclei that stained positive for VP-1 and sensitivity to anti-viral treatments [86]. The researchers also successfully cultured tubuloids derived from Wilms tumor patient biopsies [86]. Nephroblastoma tubuloids showed differing morphologies, supported survival of stromal and non-epithelial cells, and exhibited elevated SIX2 expression as compared to healthy cells. These investigators even modified their tubuloid approach to model cystic fibrosis [86]. As a non-invasive tactic, urine was collected from patients harboring the CFTR mutation to isolate renal tubular epithelial cells. Incredibly, tubuloids can be grown from these urine-derived patient cells and display disease hallmarks such as reduced luminal chloride transport. Lastly, to scale-up their system, healthy tubuloids were plated on an ‘organ-on-a-chip’ perfusion platform [86]. Upon plating, cells assembled polarized tubules expressing functional adherens and tight junctions. Calcein accumulation assays indicated tubules-on-a-chip perform trans-epithelial transport as fluorescence was enriched on apical surfaces. Excitingly, this functional system could enable detailed modeling of renal channelopathies and facilitate drug screening to identify personalized treatments for these patients.

Although tubuloids appear to generate more mature, functional epithelium than other kidney organoid protocols, this system does not support the study of glomerular diseases, as podocyte markers are absent. Typically, the composition of individual tubuloid units was biased in that each contained a majority of one nephron segment type. Altogether, the expression of loop of Henle-specific markers (UMOD, SLC12A1) and distal tubule markers (CALB1) were underrepresented [86]. To address these shortcomings, methods described by Glass et al., 2020 could be tested to determine if particular WNT and FGF9 regimens can shift tubuloid populations to a more distal identity [90]. Although the tubuloid system has certain drawbacks, it provides an efficient pipeline that can realistically be applied in the clinic to create personalized models of renal diseases.

## 7. Drug Screening to Discover Therapeutics and Perform Nephrotoxicology Studies

Drug development is immensely challenging, requiring a huge investment of time and resources, and is plagued by a high failure/attrition rate even for the very best candidate molecules that make it all the way to preclinical or clinical trials [138]. Advances in generating renal organoids to achieve enhanced differentiation and/or maturation characteristics are providing some new vistas for drug screening with these systems, but the path is still littered with caveats and limitations.

Several recent studies have created simple, cost-effective, scalable systems for drug discovery. For example, proximal tubule ‘enhanced’ organoids express and properly localize a suite of solute transporters, unlike standard kidney organoids [110]. Indeed, when tested for their functionality, they were found to uptake albumin, exhibit the capacity for organic cation uptake, and have higher expression of cisplatin transporters [110]. In 2022, Tran et al. reported a platform for modeling autosomal dominant polycystic kidney disease (ADPKD), where they induced genetic mutations in PKD1 and PKD2 in human pluripotent stem cells [139]. After about 2 weeks of culture, the mutant organoids formed cystic structures, and the researchers used mass production strategies to generate 96-well plates containing methylcellulose-embedded organoids for high-throughput screening. Several expected cyst inhibitors scored as hits in their approach, and they identified a drug named quinazoline as a novel inhibitor as well, showing an important proof-of-principle for this experimental pipeline.

In another 2022 study, Xu et al. published a study in which renal tubuloids were used to model ADPKD with a similar gene-editing approach used to disrupt *PKD1* and *PKD2*, with cyst formation occurring within 10 days [140]. Here, the researchers tested the efficacy of tolvaptan, which is the only FDA-approved compound that reduces cyst growth and disease progression in ADPKD [141,142,143]. They observed a time-dependent effect of tolvaptan treatment on cyst size only in *PKD1*^−/−^ and *PKD2*^−/−^ tubuloids that were derived from a distinct subpopulation of CD24+ human kidney cells, which are hypothesized to represent an adult renal stem/progenitor population [144,145,146,147]. Similar effects were not observed with human pluripotent stem cell-derived organoids [140]. Further work is needed to understand these effects, specifically, the tubule segment(s) they may be pertinent to, as the tubuloids in this study ectopically expressed the vasopressin receptor AVRP2, which is the primary target of tolvaptan [140].

In sum, kidney organoids pose a number of significant limitations for current disease modeling due to features such as their early/fetal developmental character and off-target cell types. Despite these limitations, however, the components of 3D culture systems like renal organoids provide powerful opportunities to evaluate drug nephrotoxicity because of the paucity of complex in vitro cell models for the kidney [148]. Until now, the latter consisted of two-dimensional (2D) cell culture, which is incapable of recapitulating the kidney’s unique structural arrangements and its physiology. Nephrotoxicology studies using renal organoids now include drugs such as aspirin, cisplatin, and aristolochic acid [50,57,67,72,82,149,150,151,152,153]. This list will most certainly continue to expand.

## 8. Future Directions

**Figure 2:**
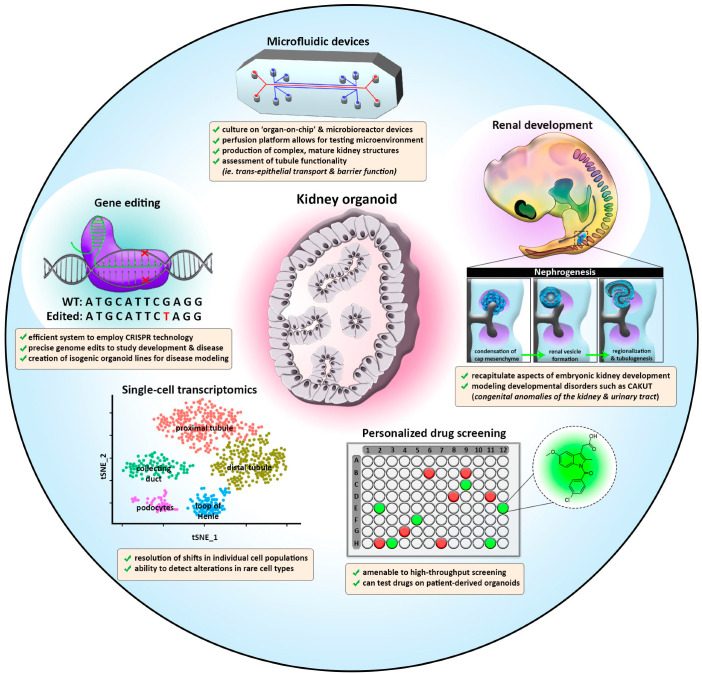
Diagram illustrating evolving applications of kidney organoid technologies. Microfluidic devices (**top**) create a platform for assessment of renal function and may enhance the differentiation of tubule epithelium due to continuous perfusion of secreted factors. Growing kidney organoids in vitro mimics processes of renal development (**top right**), like nephrogenesis, and can serve as a model for future studies on the molecular and cellular changes which transpire in congenital kidney diseases. Kidney organoids are amenable to personalized drug screening (**bottom right**) and may facilitate the discovery of novel treatment options as well as provide a much-needed platform for nephrotoxicology studies. The advent of single-cell transcriptomics (**bottom left**) has assisted the optimization of kidney organoid protocols and can help detect shifts in specific cell populations in disease contexts via clustering analysis. Kidney organoid models are an effective system to apply genetic engineering strategies (**top left**), like CRISPR-Cas9. The ability to produce precise genetic alterations and isogenic organoid lines will catalyze future discoveries concerning kidney development and disease.

The creation of kidney organoid protocols offers many exciting prospects for future research and for the ultimate goal of ‘re-building’ a kidney [154] (Figure 2). While present kidney organoid protocols manage to achieve early tissue patterning, they currently still struggle to support continued maturation, proliferation, and growth. This review has explored a number of trailblazing studies in kidney organoids, with a focus on how these systems are adapting to accurately model development and disease processes. Indeed, there is a steadily expanding literature on renal organoid disease models.

With the dawn of single-cell RNA sequencing and microbioreactor platforms, it is possible to grow renal organoids in vitro that are increasingly similar to human kidneys (Figure 2). The continued application of these methods will likely yield increasingly more faithful organoid systems to better model development and human disease and employ for precision medicine.

The optimization of culture conditions that allow the perfusion of paracrine signaling and the application of signaling molecules at the correct dose and time will facilitate the expansion and further maturation of kidney organoids. Renal organoids derived from either iPSCs or ASCs both possess great potential to address major questions in the field pertaining to embryogenesis and disease mechanisms. This is an exciting time, as collectively, these initiatives bring to light a versatile model that is continually evolving to recapitulate higher-order kidney structures and can pave the way for personalized disease modeling. In the sections below, we explore several future prospects.

### 8.1. Generating Higher-Order and Vascularized Kidney Organoids While Reducing Heterogeneity

Researchers have devised several ingenious strategies to produce large numbers of renal organoids using engineering techniques. “Bioprinting” is an automated method that has been used to rapidly generate renal organoids with the same size and cellular composition. When generated by three-dimensional bioprinting, renal organoids impressively exhibit less heterogeneity than structures generated via “manual” techniques [100]. This manufacturing approach increased production ninefold, and researchers designed systems for fabrication in various multi-well formats. Of these, the ability to fabricate in a 96-well plate format, with one renal organoid per well, provides a convenient strategy for screening chemical libraries with automated approaches. This provides many possibilities for future research, and it is likely that the continued application of bioengineering strategies will play an important role in the years to come [155].

### 8.2. CRISPR Screening: A Promising Quest to Find Mechanisms of Human Renal Development

The advent of CRISPR-Cas9 protocols has led to powerful ways to perturb genetic pathways and screen for relevant phenotypes [156,157,158,159,160]. CRISPR-Cas9 is a high throughput tool that rapidly edits the genome. To target the region of interest, sgRNAs are specifically developed and complexed with the Cas9 enzyme to introduce a double-strand break. While a simple and effective method in most organisms and 2D cultures, utilizing CRISPR-Cas9 in 3D organoids has posed a unique challenge to the field. The complexity of varying proliferative rates in organoids can cause differing levels of sgRNA, often inaccurately representing the functionality of a gene.

Ungricht et al. recently overcame this obstacle by conducting a genome-wide screen with inducible Cas-9 in nephron organoids [106]. In this study, temporal control of gene editing allowed researchers to specifically interrogate the difference between lineage specification and maturation. To screen phenotypes, they used an epithelial cell surface marker (EPCAM) to detect tubular epithelial cells. Overall, the study found a plethora of gene hits for developmental pathways in the kidney, including Rho-associated protein kinase (ROCK), NOTCH ligand JAG1, and genes associated with congenital anomalies of the kidney and urinary tract (CAKUT). This dataset provides a large reserve of information on both known and novel genetic pathways for future researchers to further investigate. The ability to edit the genome of kidney organoids will allow for research into other renal cell types, analysis of congenital diseases, and continued elucidation of genetic patterning pathways that remain to be understood.

### 8.3. Growing an Expanded Repertoire of Genetic Disease Models

Kidney afflictions impact at least 10% of the world’s population, and many remain poorly understood. [161]. Renal organoids provide one avenue through which to close this gap by modeling genetic diseases that affect the kidney, such as ciliopathies [162]. While several of the tubular genetic disease models have been discussed in previous sections, there are numerous proof-of-principal studies in which glomerular genetic diseases have been successfully modeled as well. For example, the knockout of the gene PODXL in hiPSCs led to renal organoids with alterations in podocyte structure [58]. Further, in 2018, Hale et al. used cells isolated from a patient with congenital nephrotic syndrome that harbored mutations in the *NPHS1* gene to derive kidney organoids [72]. The podocytes in the cultures exhibited similar alterations in foot process extensions and gene expression, as seen in patients with this podocytopathy [72]. More recently, renal organoids were cultured from a patient with *NPHS2* mutations and were thus used to model congenital nephrotic syndrome [163]. In related work, a series of hiPSCs with pathogenic mutations in *NPHS2* were generated and used to elucidate the effect of each variant on protein trafficking [164].

There are also renal conditions that have been difficult to study in animal models, such as genetic defects in *APOL1*. Mutations in *APOL1* are associated with high CKD risk, but the reasons for these pathologies are largely mysterious. To address this, Liu et al. used CRISPR-Cas9 genome editing of hiPSCs to knock in deleterious variants, thereby creating a useful platform to study the consequence of these variants within renal organoids [165].

### 8.4. Renal Organoids and Precision Medicine

Kidney organoids provide a long sought-after source of human patient-specific renal tissue that opens a new chapter in basic research and clinical nephrology. They can be used to explore specific treatments for kidney diseases, thus allowing for many innovative prospects in the future of personalized medicine. Isolation of adult renal progenitor cells from the urine of patients affected by genetic kidney disorders paved the way to use this source in generating 3D kidney structures to make the diagnosis of genetic kidney disease [166]. In the dawning era of personalized medicine, the prospect of cultivating and observing patient-derived renal organoids can provide new insights into the molecular features affecting an individual’s own native kidney. These observations could be used to guide the selection and formulation of patient-tailored therapeutics.

Renal organoids will likely expected to play a major role in the quest for cell therapy and renal replacement strategies to treat kidney failure. Because hiPSC organoids mimic fetal nephrogenesis, they are especially well suited for modeling developmental diseases but have also been used for conditions like diabetes [167]. Tubuloids are particularly promising for studying the responses to acute injury [168,169,170]. In addition, as they can be easily and rapidly established from autologous cells with high efficiency, they offer the option to generate large biobanks and facilitate personalized medicine with relative ease. Tubuloids are also suited to study diseases that manifest in the fully developed kidney, such as renal cancer [171] and infectious diseases [172].

## 9. Conclusions

Renal organoids have captured the imagination of many scientists, clinicians, and the public as well. These amazing miniature structures have immense allure, proffering novel opportunities to (1) understand human-specific aspects of organ biology, whether it be the fundamental developmental mechanisms, function, disease etiology, and/or pathology; and (2) therapeutic potential from the smallest scale of drug testing to the most grandiose promise of serving as autologous whole organ replacement/augmentation tools or obtaining compatible organs from “living biobanks.” With continued industry, it is our hope that renal organoids can be fully wielded as a force for good in the ongoing scientific quest to identify regenerative medicine approaches for kidney afflictions.

## Data Availability

Not applicable.
